# H1153Y-*KCNH2* Mutation Identified in a Sudden Arrhythmic Death Syndrome Case Alters Channel Gating

**DOI:** 10.3390/ijms22179235

**Published:** 2021-08-26

**Authors:** Audrey Farrugia, Kevin Rollet, Jérome Sinniger, Susana Brun, Caroline Spenle, Bertrand Ludes, Omar Taleb, Ayikoe Guy Mensah-Nyagan

**Affiliations:** 1 Institut de Médecine Légale de Strasbourg, Fédération de Médecine Translationnelle de Strasbourg (FMTS), Université de Strasbourg, 11 Rue Humann, 67000 Strasbourg, France; 2 Biopathologie de la Myéline, Neuroprotection et Stratégies Thérapeutiques, INSERM U1119, Centre de Recherche en Biomédecine de Strasbourg (CRBS), Fédération de Médecine Translationnelle de Strasbourg (FMTS), Université de Strasbourg, 1 Rue Eugène Boeckel, 67000 Strasbourg, France; k.rollet@ibmc-cnrs.unistra.fr (K.R.); jerome.sinniger@gmail.com (J.S.); susana.brun@unistra.fr (S.B.); cspenle@unistra.fr (C.S.); omar@unistra.fr (O.T.); gmensah@unistra.fr (A.G.M.-N.); 3 Institut de Médecine Légale de Paris, Université de Paris, F-75012 Paris, France; bertrand.ludes@parisdescartes.fr; 4 Université de Paris, BABEL, CNRS, F-75012 Paris, France

**Keywords:** electrophysiology, Kv11.1 channel, long QT syndrome 2, sudden arrhythmic death case, current density

## Abstract

Long QT syndrome is one of the most common hereditary channelopathies inducing fatal arrhythmias and sudden cardiac death. We identified in a sudden arrhythmic death syndrome case a C-term *KCNH2* mutation (c.3457C > T; p.His1153Tyr) classified as variant of unknown significance and functional impact. Heterologous expression in HEK293 cells combined with western-blot, flow-cytometry, immunocytochemical and microscope analyses shows no modification of channel trafficking to the cell membrane. Electrophysiological studies reveal that the mutation causes a loss of HERG channel function through an alteration of channel biophysical properties that reduces the current density leading to LQT2. These results provide the first functional evidence for H1153Y-*KCNH2* mutation-induced abnormal channel properties. They concur with previous biophysical and clinical presentations of a survived patient with another variant that is G1036D. Therefore, the present report importantly highlights the potential severity of variants that may have useful implications for treatment, surveillance, and follow-up of LQT2 patients.

## 1. Introduction

Ion channels play a crucial role in generating membrane potential and function in several cellular activities such as signal transduction, neurotransmitter release, hormone secretion, or muscle contraction including the heart myocardium [1]. The cellular action potential driving the heart cycle is shaped by specific series of depolarizing and repolarizing ion currents mediated by ion channels. Hereditary cardiac channelopathies are a set of various electrical disorders in structurally normal hearts that leave the myocardium vulnerable to instability and predisposition to life-threatening ventricular arrhythmias [2]. These disorders most commonly respond to mutations in genes encoding cardiac ion channels or receptors and/or their regulatory proteins and follow predominantly an autosomal dominant pattern of inheritance [3]. The major cardiac channelopathies that could induce sudden cardiac death (SCD) are long QT syndrome (LQTS), short QT syndrome (SQTS), Brugada syndrome (BrS), and catecholaminergic polymorphic ventricular tachycardia (CPVT) [4,5].

In the last decade, the search for specific causes of the inheritable risk of SCD was boosted by the availability of high-throughput sequencing technologies that made possible the targeting sequencing approach, whole-exome sequencing, or genome-wide association studies in large cohorts of sudden arrhythmic death syndrome (SADS). Precisely, the term SADS encompasses cases with no established cause of death after autopsy and toxicological and histological analyses [6,7,8,9,10]. Despite the breakthrough and great technological advance allowed by high-throughput sequencing approaches for the systemic characterization of mutations, the clinical interpretation of the identified genetic variants remains a major challenge that is not yet resolved. This is particularly true for SADS-related diseases, which are characterized by incomplete penetrance and variable expressivity [11], and because most SADS cases did not undergo a premortem electrocardiogram ECG. Especially in the field of channelopathies with the risk of sudden death, an additional problem is the uncertainty of the previously reported pathogenicity of some variants, which most likely are not pathogenic, as it was evidenced in some reports [12]. For example, a study indicated that 30% of disease-causing genetic variants cited in the literature are common polymorphisms or misinterpreted variants [13].

In a previous postmortem genetic study, we encountered difficulties in evaluating the pathogenicity of the variant c.3457C > T (p.His1153Tyr) detected in the cardiac potassium channel gene *KCNH2,* identified in a 20-year-old man who died during a bicycle exercise in a fitness room [14]. The variant of interest was identified in the *KCNH2* gene (*human ether-a-go-go related gene or hERG*) [15,16], which encodes the voltage-gated K^+^ channel alpha-subunit Kv11.1. Four Kv11.1 alpha subunits co-assemble in a tetrameric ion channel that conducts the rapidly activating delayed rectifier K^+^ current in the heart (Kr) [16,17]. Each homologous subunit is composed of six transmembrane segments with intracellular N and C terminal domains.

The loss-of-function *KCNH2* mutations decrease IKv11.1 or IKr in type 2 LQTS (LQT2), which is characterized by a prolonged duration from ventricular depolarization to repolarization that corresponds to the QT interval on an ECG.

Almost 500 *KCNH2* putative mutations have been linked to LQT2 and have been classified into four molecular mechanisms: class 1, abnormal transcription/translation; class 2, deficient protein trafficking; class 3, abnormal channel gating/kinetics; and class 4, altered channel permeability [18]. About 60% of LQT2-linked *KCNH2* mutations are missense and cause a loss of function by disrupting either channel trafficking to the cell membrane (class 2 mechanism), the gating (class 3 mechanism), or the single channel current (class 4 mechanism).

Over 150 suspected LQT2-causing missense mutations have been studied using heterologous expression systems, and these demonstrate that 88% of LQT2-linked missense mutations disrupt the Kv11.1 channel function via a class 2 mechanism, 5% alter gating, 2% alter permeation, and 6% are benign [19].

In the present study, we combined several technological approaches to evaluate the functional impact of the H1153Y-*KCNH2* missense variant transfected into a HEK293 heterologous expression system. We tested the hypothesis that this variant may induce a loss of function of the Kv11.1 protein channel by reducing its intracellular trafficking to the cell membrane and/or by affecting the channel gating properties.

## 2. Results

### 2.1. Mutation Analysis

**Figure 1:**
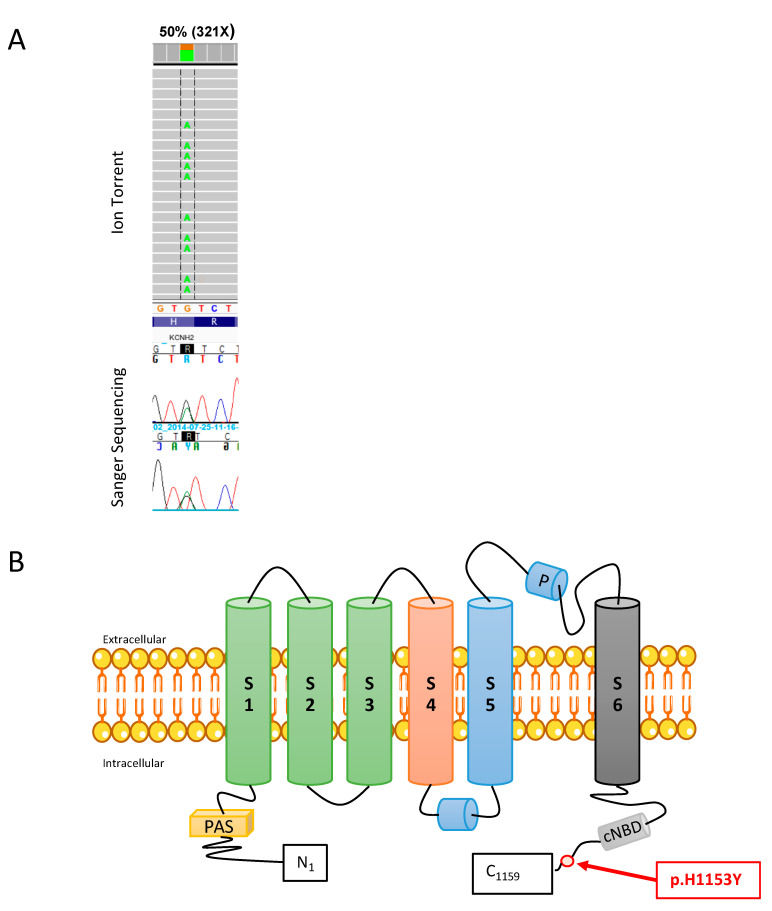
Molecular position of the *KCNH2* H1153Y variant (NM_0002383; rs 199473035) detected using the Ion torrent^TM^ PGM platform and confirmed with Sanger sequencing. (**A**) The reads of NGS sequencing and the DNA sequence chromatogram illustrate the heterozygous c:3457C > T nucleotide substitution that results in the substitution of a Histidine-H (positively charged amino acid) for a Tyrosine-Y (polar uncharged amino acid) at amino acid residue 1153. (**B**) Cartoon of one Kv11.1 channel subunit. The circle denotes the approximate location of the p.H1153Y variant in the C-term region.

Genetic analysis using targeted NGS identified a *KCNH2* missense variation (NM_0002383; c.3457C > T, rs 199473035), confirmed by direct sequencing with an automated capillary sequencer (Figure 1A). This mutation results in the substitution of the amino acid Histidine (positively charged amino acid) at position 1153 by Tyrosine (polar uncharged amino acid). This residue is localized in the C-term region of the protein (Figure 1B), which may be involved in multiple aspects of channel physiology such as subunit assembly, trafficking, and functional regulation [20,21]. Indeed, the *KCNH2* H1153Y variant was previously reported in LQTS patients by Napolitano et al. in 2005 [22] and classified as class 3 (variants of unknown significance, VUS) according to ACMG guidelines.

Table 1 summarizes the amino acid substitution effect prediction methods (Mutation Tester, Sorting Invariant from Tolerated-SIFT, Polyphen-2), allele frequency in the Genome Aggregation Database (GnomAD), and the literature cited by the Human Gene Mutation Database (HGMD) [23]. However, the H1153Y-Kv11.1 channel has not been functionally characterized so far.

### 2.2. H1153Y-Kv11.1 CHANNEL Proteins Have Similar Trafficking Phenotype to WT-Kv11.1 Channel Proteins: Similar Surface Expression and Glycosylation Level

To determine the trafficking phenotype of H1153Y-Kv11.1 channel proteins, we combined immunostaining, biochemical assay with sensibility test to Proteinase K (pK), and flow cytometry assessment, previously validated as robust and sensitive to quantify the surface expression of Wild Type (WT) and LQT2 Kv11.1 channel variants [25].

**Figure 2:**
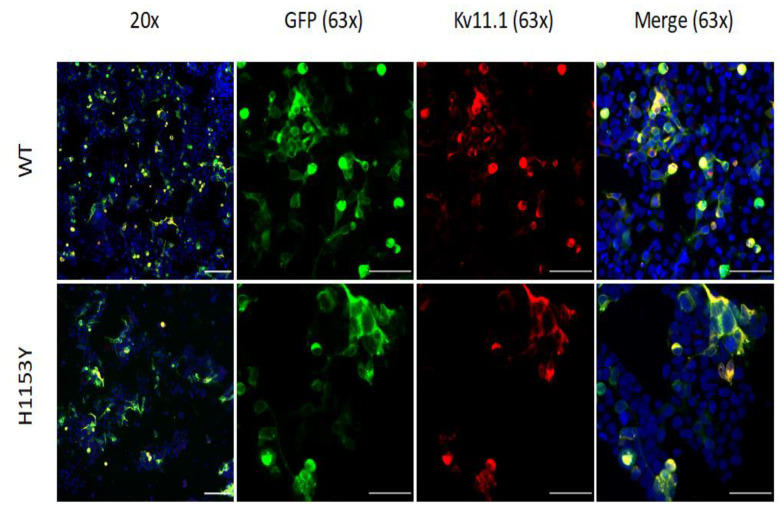
Representative microscopy images of HEK293 cells expressing WT-Kv11.1 and H1153Y-Kv11.1. GFP fluorescence is shown in green. Anti-Kv11.1 extracellular immunostaining (Alexa 647 fluorescence) is shown in red. Overlapping immunostaining of red and green signals of similar intensities is shown as yellow, and the cell nuclei are labeled blue. The scale bar represents 100 µm at 20× magnification and 50 µM at 63× magnification.

Immunocytochemical analysis in permeabilized cells was performed to explore the overall cellular expression and localization of WT and H1153Y channels. The control cells, corresponding to HEK293 transiently transfected with pcDNA3.1-NeGFP, have green background fluorescence. The cells expressing pcDNA3.1-NeGFP-WT-Kv11.1 or pcDNA3.1-NeGFP-H1153Y-Kv11.1 protein had no difference in the immunostaining pattern at the plasma membrane and cytoplasm level (Figure 2).

The microscope imaging allowed for cellular/subcellular visualization but did not provide the ability to easily quantify a large number of cells in an unbiased fashion.

Hence, we performed flow cytometry studies that enabled rapid analyses of a large population of cells with single-cell resolution and allowed the quantification of channel surface and total expression. The enhanced GFP-tagged Kv11.1 construct (eGFP is fused to the N-terminus of Kv11.1) enabled simultaneous fluorescence detection of total Kv11.1 expression (GFP in green) and surface Kv11.1 expression with anti-Kv11.1 extracellular antibody (Alexa 467 fluorescence) in non-permeabilized cells.

**Figure 3:**
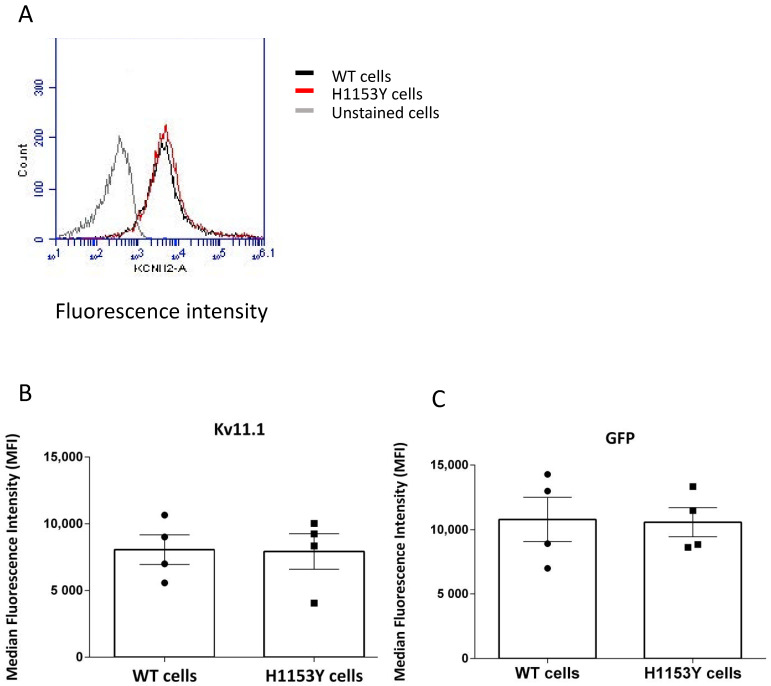
Flow cytometry qualitative (**A**) and quantitative (**B**,**C**) assessment of WT-Kv11.1 and H1153-Kv11.1 channel expression. (**A**) Representative histograms for unstained cells (corresponding to non-transfected cells) and cells transiently transfected with WT-Kv11.1 and H153Y-Kv11.1 stained with antiKv11.1 conjugated antibody. (**B**) Quantification of surface Kv11.1 channel (Alexia 647 fluorescence) for WT and H1153 variants in non-permeabilized cells. (**C**) Quantification of total Kv11.1 channel (GFP fused to the N-terminus of Kv11.1) for WT and H1153-Kv11.1 in non-permeabilized cells. The Median Kv11.1 Intensity of Fluorescence (MIF) represents the median of fluorescence intensity of 10,000 cells counted for each experiment (*p* > 0.05). Each value represents the median+ S.E.M. of four independent experiments.

The quantification of surface (Figure 3A) and total (Figure 3B) median fluorescence intensity from four independent experiments revealed a similar protein distribution, and no difference between WT and H1153Y-Kv11.1 channel surface and total expression was observed (Figure 3C).

**Figure 4:**
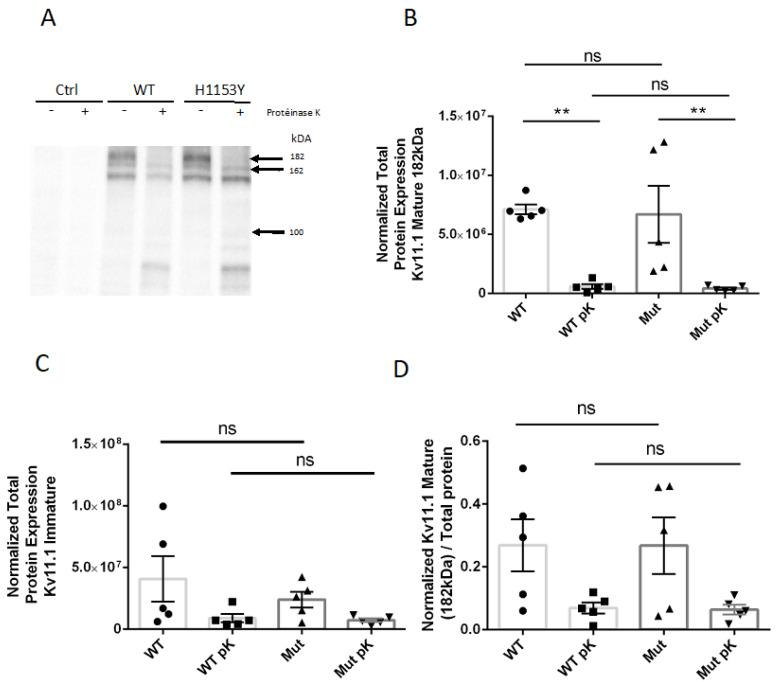
Trafficking phenotype of WT-Kv11.1 and H1153-Kv11.1 channel protein. (**A**) Representative Western blot analyses of HEK293 cells transiently transfected with pcDNA3.1-N-eGFP (control cells), WT-Kv11.1, or H1153-Kv11.1, with or without pK treatment (200 μg/mL, 30 min); Kv11.1 channel protein was detected with an anti-Kv11.1 antibody targeting intracellular domain. Kv11.1 WT and H1153 showed two bands corresponding to a mature complex-glycosylated 182 kDA Kv11.1 band (155 kD + 27 kDA of GFP) completely digested by pK and one band at slightly lower molecular weight (162 kD) resistant to pK cleavage corresponding to immature core-glycosylated Kv11.1 protein band. (**B**–**D**) Semi-quantification of mature (**B**) and immature proteins (**C**) in WT and H1153Y cells with and without pK. Total protein measured with stain-free method was used to normalize, n = 5 independent experiments (** (*p* < 0.01) = for difference between WT and pK-treated WT, and for difference between mutant and pK-treated mutant; ns = no significant difference (*p* > 0.05) between mutant and WT. (**D**) The relative amount of mature Kv11.1 protein (mature/total Kv11.1) based on immunoblot analyses for each set of experiments is plotted (*p* > 0.05). The error bars represent SEM.

To explore the effects of H1153Y-*KCNH2* mutation on the mature and immature Kv11.1 protein expression, we took advantage of the two separate bands on the immunoblot (135 kD and 155 kD bands) (Figure 4A). The Kv11.1 protein (132 kD) is initially synthesized in the endoplasmic reticulum (ER), where it undergoes N-linked core-glycosylation to form immature channel proteins (subunit MW: 135 kD core-glycosylated form). The immature proteins are then trafficked to the Golgi for N-linked complex glycosylation to become fully glycosylated mature proteins (subunit MW: 155 kD) that are predominantly at the cell surface [26,27]. Consequently, biochemical analyses of the relative abundance of 135 and 155 kDa Kv11.1 bands are a standard widely adopted tool to analyze the trafficking of WT and mutant Kv11.1 channels under different conditions [28].

To study the cell surface expression of WT and H1153Y-Kv11.1 channel proteins, and to evaluate more precisely their subcellular distribution of mature and immature forms, WT and H1153Y-expressing cells were exposed to pK to digest the domains of Kv11.1 protein on the cell surface. Consistently with previous observations [27,29], WT-Kv11.1 expressed two protein bands in our conditions: an upper band—182 kDa (155 kDa + 27 kDa of GFP fused to Nter), and a lower band—162 kDa (135 kDa + 27 kDa of GFP fused to Nter). A third band of less than 150 kDa was also visible on the immunoblot like in the study of Smith et al. [30], and may correspond to a cleaved form of the protein. The upper band, corresponding to the mature protein, disappeared almost completely with pK treatment for WT (P = 0.0079) and H1153Y-expressing cells (*p* = 0.0079) (Figure 4B), which confirmed a predominant transmembrane form of the channel. This was associated with the appearance of lower molecular bands of 60–75 kDa, as previously described [27]. The intensity of the lower band was not statistically different between WT and H1153Y cells (*p* > 0.05). After pK treatment, the lower band had a slightly decreased intensity in some experiments, which may indicate the presence of an immature channel at the cell surface [31], although this decrease was not statistically significant and similar mean intensity values were obtained before and after pK treatment (Figure 4C). The WT-Kv11.1 sensitivity to pK treatment was not significantly different to that of the variant H1153Y-Kv11.1 (Figure 4A–C), and thus the ratio of the relatively mature H1153Y-Kv11.1 protein showed no statistical difference with WT-Kv11.1 (Figure 4D).

These Western blot data with semi-quantitative analyses demonstrated that H1153Y-*KCNH2* mutation did not induce the modification of Kv11.1 channel trafficking.

### 2.3. Electrophysiological Characterization of the Mutation H1153Y in hERG Channel

**Figure 5:**
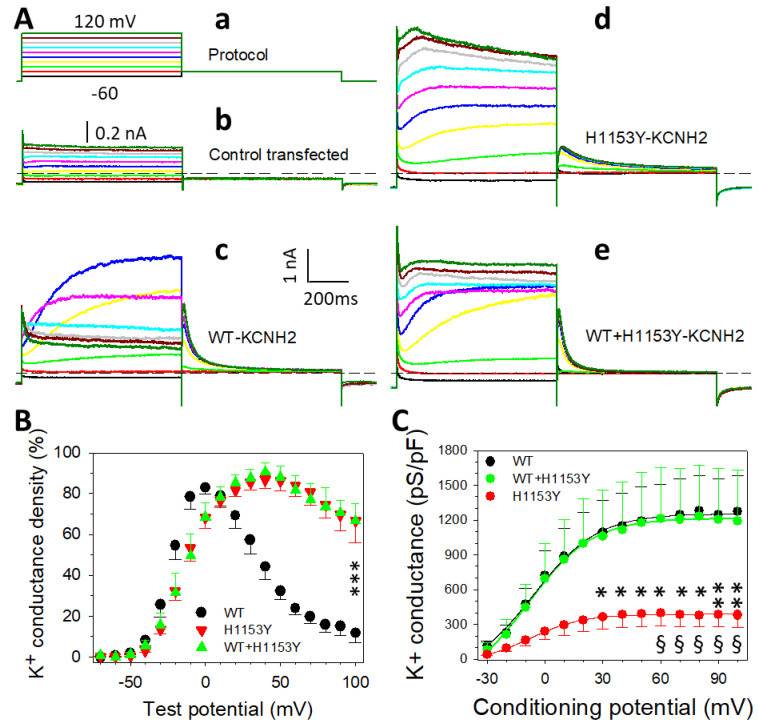
Voltage-dependent activation of WT, H1153Y, and WT + H1153Y hERG channels. (**A**) Current traces obtained using the protocol given in (**a**) for the control (**b**), WT (**c**), H1153Y (**d**), and WT + H1153Y (**e**). The dashed lines indicate the zero-current level. (**B**) Mean activation distributions of the steady-state current measured at the end of the depolarizing steps, obtained from WT (n = 16 cells), H1153Y (n = 20 cells), and WT + H1153Y (n = 8 cells) expression conditions as indicated. (**C**) Mean G–V relations of the deactivation current recorded for WT, H1153Y, and WT + H1153Y as indicated. Continuous curves are best fitted curves to data of the Boltzmann equation obtained with parameters Gm = 1405, 418, and 1467 pS/pF, V_1/2_ = −6.1, −8.0, and −8.7 mV, and k = 16.7, 13.8, and 16.6 for WT, H1153Y, and WT + H1153Y, respectively. * = comparison of WT vs. H1153Y and § = comparison of H1153Y vs. WT + H1153Y.

The H1153Y-*KCNH2* variant was characterized and compared to the WT-*KCNH2* variant using the HEK293 cells’ heterologous expression system. To imitate the heterozygous situation typical in patients, co-expression of a mix of WT and H1153Y (2.63 µg of DNA of each) was also characterized and compared to each of the variants. Figure 5A illustrates typical current traces recorded from cells transfected with the empty vector (Figure 5(Ab)), WT- (Figure 5(Ac)), H1153Y- (Figure 5(Ad)), or WT + H1153Y-*KCNH2* (Figure 5(Ae)). Non-transfected HEK293 cells expressed almost no voltage-dependent channels and the I–V relationships were quasi-linear. Similarly, the I–V relation obtained from cells transfected with the vector containing only the GFP coding gene as an insert was linear; however, the mean cellular slope conductance was slightly higher (510 ± 110 pS n = 5 as compared to 310 ± 57 pS n = 22), although not statistically significant.

When HEK293 cells were transfected with the vector containing the WT-*KCNH2* or H1153Y-*KCNH2* gene encoding for the Kv11.1 or hERG channel, about 50% (n = 121 from nine different transfection experiments) of the cells tested exhibited a typical voltage-dependent I–V relationship (Figure 5(Ac–e),B,C). These results revealed the expression of a voltage-activated outward current that had an activation threshold between −50 and −40 mV (Figure 5B), and a maximal peak current occurring at about 0 and 40 mV for the WT- and H1153Y-Kv11.1, respectively, that was followed by a significant voltage-dependent inactivation for more positive test potentials. This inactivation was of different amplitudes for WT and H1153Y-Kv11.1 variants (Figure 5B). Overall, the mean inactivation proportion of the maximal current for the WT and H1153Y was different and statistically significant (*t* test comparison of means, P < 0.001; Figure 5B) at 100 mV with values of 86.0 ± 4.4 and 23.4 ± 7.0%, respectively. Co-expression of WT- and H1153Y-*KCNH2* did not rescue the decrease in inactivation observed with the mutant variant and a similar mean value of inactivation proportion was obtained (24.5 ± 8.9%). The expressed outward current had a similar reversal potential for WT, H1153Y, and WT + H1153Y, respectively, and mean values of −87.0 ± 1.0, −88.6 ± 1.5, and −86.5 ± 3.0 mV were obtained, close to the K^+^ equilibrium potential (E_K_ = −90.4 mV) in our recording conditions. These observations suggest that the outward current obtained in each expression condition was carried out by similar K^+^ ion movements.

Large deactivation or tail currents were recorded when returning to negative membrane potentials (Figure 5(Ac)) whose specific conductance–voltage (G–V) relation (Figure 5C) had no apparent voltage inactivation. However, the mutant variant H1153Y exhibited a G–V relation with parameters significantly different from those of the WT. Indeed, using the Boltzmann model, the parameters maximal activated conductance (Gm), half maximal activating potential (V_1/2_), and slope factor (k) were estimated for each individual G–V relation, and the calculated mean values had statistically significant differences for Gm (1176 ± 288 and 431 ± 115 pS/pF; *p* < 0.01). Figure 5C illustrates the mean G–V relationship obtained in each expression condition (i.e., WT-, H1153Y-, and WT + H1153Y-Kv11.1). The other parameters for the WT and H1153Y variant, V_1/2_ (−2.2 ± 2.9 and −0.3 ± 2.7 mV, respectively) and k (13.5 ± 1.5 and 12.5 ± 1.1, respectively), were not statistically different. The co-expression of WT and H1153Y rescued the maximal deactivation K^+^ conductance to a value close to that of the WT (Figure 5C) and different from the value of the H1153Y variant (*p* < 0.05). Mean parameter values obtained for the co-expression were Gm = 1369 ± 319 pS/pF, V_1/2_ = 4.6 ± 3.6 mV, and k = 14.2 ± 1.6.

**Figure 6:**
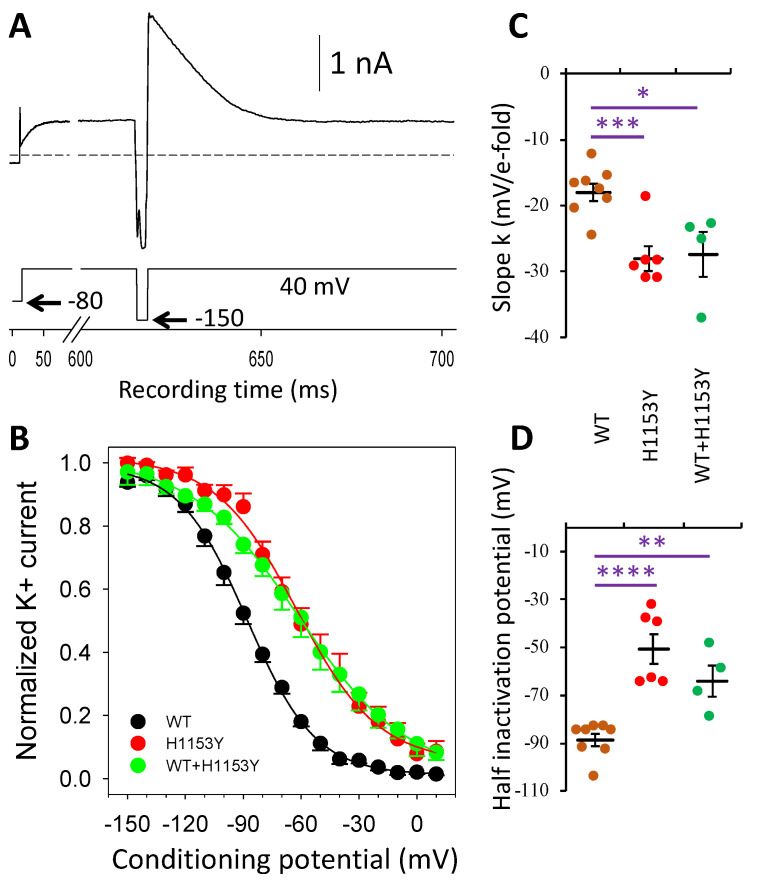
Fast recovery from voltage inactivation of the WT, H1153Y, and WT + H1153Y hERG channels. (**A**) Protocol (lower trace) used and current response (upper trace) obtained from a WT-*KCNH2* transfected HEK293 cell. The dashed line represents the zero-current level. (**B**) Normalized mean voltage-dependent inactivation distributions for WT (n = 8 cells), H1153Y (n = 6 cells), and WT + H1153Y (n = 4 cells). Continuous curves are best fit of the Boltzmann equation to data points with parameters of maximal normalized current Im = 0.98, 0.96, and 1.00, half inactivation potential V_1/2_ = −88.0, −62.2, and −61.2 mV, and a slope factor k= −17.7, −21.1, and −28.0, respectively. (**C**,**D**) Individual data point distribution and mean (±SEM) data for k (**C**) and V_1/2_ (**D**) parameters obtained from individual voltage-dependent inactivation distributions. Comparison of WT and WT + H1153Y vs. H1153Y: asterisks denote significant difference (* *p* < 0.05; ** *p* < 0.01; *** *p* < 0.001; **** *p* < 0.0001).

Fast recovery from inactivation was evaluated using the protocol illustrated in Figure 6A (lower trace). Following a 600 ms depolarizing step potential to 40 mV, reactivating potential steps from 10 to −150 mV for 3 ms duration resulted in a deactivation current of increasing amplitude when returning to 40 mV. The I–V relationship (Figure 6B) of this current had a half inactivation potential that was different for WT and H1153Y (Figure 6B,C). Indeed, the estimated V_1/2_ from individual I–V distributions gave mean values for the WT and H1153Y variant of −88.7 ± 2.6 and −50.8 ± 6.2 mV, respectively, which was statistically different (*p* < 10^−5^). The slope factor (k) of the I–V curves also differed (*p* < 0.0007) and mean values of −18.0 ± 1.3 and −28.1 ± 1.9 were obtained, respectively. The co-expression of WT and the mutant H1153Y did not restore the alterations induced by the mutation (Figure 6C), and mean parameter values were −64.1 ± 6.5 mV and −27.4 ± 3.4 for V_1/2_ and k, respectively.

**Figure 7:**
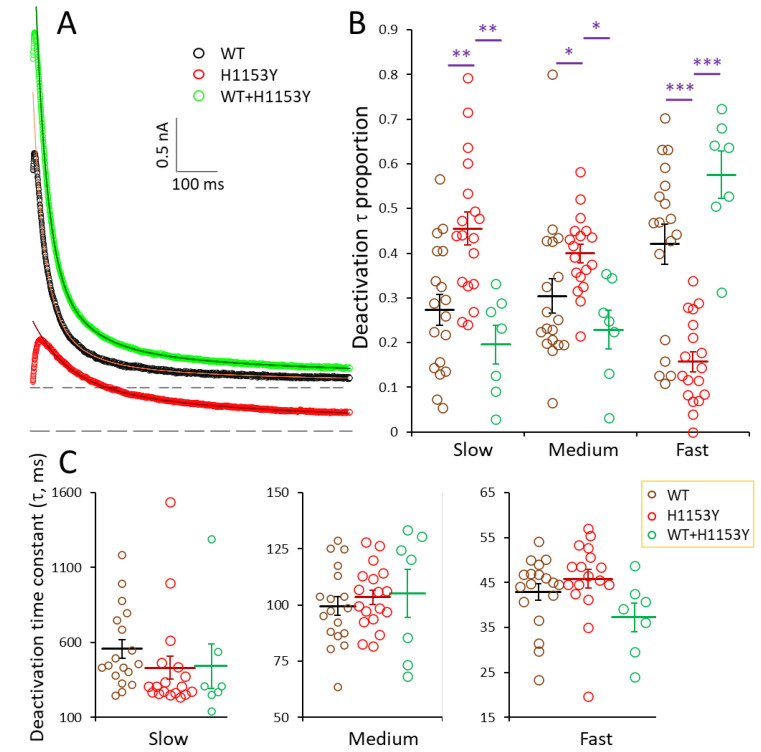
Deactivation current kinetics of the WT, H1153Y, and WT + H1153Y hERG channels. (**A)** Tail-current traces obtained at −40 mV following a depolarizing step to 40 mV for WT, H1153Y, and WT + H1153Y as indicated. Dashed lines indicate the zero-current levels for WT and WT + H1153Y (double dashed) and H1153Y (simple dashed) traces. Continuous curves are best fit of the sum of standard exponential functions obtained with 3, 2, and 3 components to the WT, H1153Y, and WT + H1153Y traces, respectively, with parameters of A_1_ = 200, 334, and 274 pA and τ_1_ = 360, 372, and 509 ms, A_2_ = 601, 734, and 779 pA and τ_2_ = 73, 82, and 93 ms, and A_3_ = 1064 and 1395 pA and τ_3_ = 24.8 and 26.9 ms, respectively. (**B**,**C**) Distribution of individual data points and mean (±SEM) for the slow, medium, and fast components (**C**) and their proportion (**B**) obtained for WT, H1153Y, and WT + H1153Y, respectively, as indicated. Comparison of WT and WT + H1153Y vs. H1153Y using one-way ANOVA followed by Newman–Keuls multiple comparison test: asterisks denote significant difference (* *p* < 0.05; ** *p* < 0.01; *** *p* < 0.001).

Following a depolarizing step to 40 mV, the individual deactivation or tail current at −40 mV in our conditions had kinetics that were best fitted by the sum of two to three standard exponentials (Figure 7A) whose time constant respective distributions were not different (one-way ANOVA, *p* > 0.4) for WT, H1153Y, and WT + H1153Y co-expression (Figure 7C). Mean time constants for the slow, medium, and fast components obtained for WT, H1153Y, and WT + H1153Y were 557 ± 62, 431 ± 78, and 444 ± 148, 99 ± 4, 103 ± 3, and 105 ± 11, and 43 ± 2, 46 ± 2, and 37 ± 3 ms, respectively. In contrast, the proportions of the kinetic components were significantly different (one-way ANOVA, *p* < 0.0001) for the WT and the mutant, the fast component proportion being reduced by a mean factor of 2.7 for the H1153Y variant (Figure 7B). The co-expression of both variants rescued this alteration, and the proportion of the fastest component was restored to a mean value similar to that obtained for the WT (Figure 7B) and significantly different (one-way ANOVA, *p* < 0.0001) from the value for the H1153Y variant.

**Figure 8:**
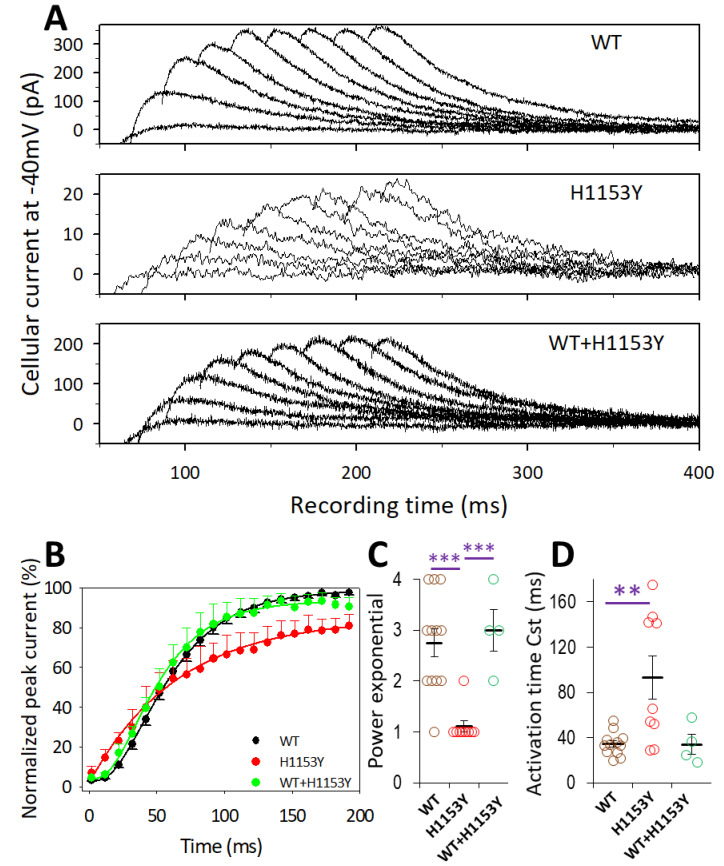
Activation time course for WT, H1153Y, and WT + H1153Y hERG channels. (**A**) Deactivation current traces obtained at −40 mV following a depolarizing step potential of duration from 22 to 182 ms by 20 ms increment obtained from cells transfected with WT-, H1153Y-, or WT + H1153Y-*KCNH2* as indicated. (**B**) Normalized peak amplitude of the deactivation current plot vs. depolarizing pulse duration for WT (n = 11 cells), H1153Y (n = 8 cells), and WT + H1153Y (n = 4 cells) as indicated. Continuous curves are best fit of exponential power function to the respective data points with parameters exponential power a = 3, 1, and 3, normalized exponential amplitude A = 98.6%, 85.7%, and 93.1%, and time constant τ = 34.1, 69.8, and 29.3 ms, respectively. (**C**,**D**) Statistical data for power exponential and time constant parameters obtained from individual time course distributions for the different expression conditions as indicated. Comparison of H1153Y vs. WT: asterisks denote significant difference (** *p* < 0.01; *** *p* < 0.001).

Figure 8A illustrates typical current traces obtained for the activation time course determination using a protocol of peak current envelope at −40 mV following a depolarizing step to 40 mV of variable duration. The peak current amplitude distributions were best fitted by a single exponential power equation (Figure 4B). For WT distributions, the best fitted function had a typical exponential power of 2 to 4 (mean value 2.8 ± 0.3, 3 for the mean distribution given in Figure 8B). In contrast, for those from the mutant, the power was typically 1 with a mean value of 1.1 ± 0.1, which was significantly different (*p* = 1.1022 10^−4^) from that obtained for the WT. This result suggests the presence of a cooperative mechanism in the activation process of WT hERG channels, while in the H1153Y variant this process declined. The activation time constant also changed and was significantly (*p* < 0.0013) slower for the mutant (Figure 8C). Mean time constant values of 34.4 ± 3.0 and 92.9 ± 19.2 ms were obtained for the WT and H1153Y, respectively. The co-expression of WT and H1153Y restored the suggested cooperative mechanism so that the best fit was obtained with a power of 2 to 4 (mean value of 3.0 ± 0.4, significantly different from that for H1153Y, *p* < 10^−4^) and the time constant to the control level (34.1 ± 8.8 ms, Figure 8C).

## 3. Discussion

Mutations in *KCNH2* are closely associated with long QT syndrome, one of the most common channelopathies, which can develop into fatal arrhythmias and SCD.

Our data provide the first description of a SADS case with H1153Y-*KCNH2* mutation and importantly evidence a significant functional impact of this mutation on the channel’s biophysical properties. Indeed, by combining molecular, biochemical, immunohistochemical, electrophysiological, and biophysical investigations, the present study demonstrates that the transient expression of the H1153Y-*KCNH2* mutant channel in a heterologous expression system causes a loss of hERG channel function by altering the channel’s biophysical properties, resulting in a reduction in the current density, without apparent modification of channel trafficking to the cell membrane.

Besides the original SADS case investigated herein, the H1153Y-*KCNH2* mutation has also been described in a LQTS patient, but the functional outcome was not established [22]. A doctoral thesis written in German also reported a mutation similar to the H1153Y-*KCNH2* mutation, but this mutation was not referenced in the Human Gene Mutation Database (HGMD^®^) Professional [32]. Thus, the lack of functional validation of the H1153Y-*KCNH2* mutation has long remained a missing key proof for a relevant clinical exploitation and previously associated LQTS must be interpreted with caution [12]. Because no SADS case with H1153Y-*KCNH2* mutation has been reported previously, and since functional study targeting this mutation has never been performed in heart disease conditions, the present paper may critically shed light on the clinical relevancy of the H1153Y-*KCNH2* mutation and on its potential implications for treatment, surveillance, and follow-up in patients with hereditary cardiac channelopathies.

Based upon the fact that 90% of LQT2-associated missense mutations induce a loss of hERG channel function via a “trafficking-deficient” mechanism, we have firstly tested this hypothesis for the H1153Y variant. Our combined Western blot and flow cytometry data demonstrated that the H1153Y-*KCNH2* mutation, located at the distal C-term of the channel subunit, did not produce trafficking deficiency of the Kv11.1 channel protein. These results concur with those from Anderson et al. [18], Biliczki et al. [20], and Anson et al. [33]. Indeed, these authors successively determined the trafficking phenotype of several variants, including 11 (A913V, R1005Q, R1007H, R1033W, G1036D, L1049P, R1055Q, A1058E, L1066V, Y1078C, P1157L), three (R954C, L955V, G1036D) and four missense variants (K897T, P967L, R1047L, Q1068R) located in the distal C-term of Kv11.1. They concluded that all these variants (except L955V) had similar trafficking to the WT. Electrophysiological recording was therefore necessary to explore two other probable underlying mechanisms: Abnormal channel gating and/or kinetics (class 3) and altered channel permeability (class 4). In Table 2, we synthetized the nine C-term *KCNH2* variants reported in HGMD that traffic similarly to the WT, and we specified their electrophysiological features [18,20,33]. Among these nine variants, we observed either electrophysiological properties indistinguishable from WT channels (P967L, R1047L) or different functional phenotypes such as slower deactivation (L1049P), slower inactivation (L1066V), and faster inactivation than the WT (K897T and Q1068R). Ionic current reductions have been detected for two variants: R954C concerning an asymptomatic patient with macrolide-induced QT prolongation, and G1036D that affected a surviving SCD patient.

It is well known that *KCNH2* mutations affecting adjacent amino acids may induce different cellular phenotypes and that some variants with the worst biophysical phenotype match with a clinical asymptomatic presentation [20]. However, according to the description of two severe phenotypes (SADS in our study and survived SCD in the work of Biliczhki et al. [20]) who carried a missense variant located in close proximity associated with a significant reduction in current density, we could speculate that these types of mutations might be at greater risk of sudden death.

In the present work, the proportion of the fastest kinetic component of deactivation or tail current was significantly reduced for the H1153Y variant, and we observed an altered cooperative mechanism in the activation process of the channel compared to the WT (*p* < 0.005). These two mechanisms could explain the loss of function observed with the H1153Y variant and the reduction in current density.

Transient co-expression of a mix of WT and mutant channel mimicking the heterozygous situation, rescue the fasted kinetic component of deactivation current and the cooperative mechanism in the activation process as well as the current density. These three parameters seem to be linked together in contrast to the voltage dependent inactivation that was not restored by the WT and H1153Y co-expression. The latter alteration may subsequently lead to an accelerated repolarization of cardiomyocytes, which potentially might induce some short QT aspects as was dominantly the case for the N588K mutant. This mutation located in the external S5-Pore linker region of the hERG channel and associated with Short QT syndrome [34,35] presents, interestingly, some similarities with H1153Y mutation. These similarities include impaired inactivation in the activation current (Figure 5B), a diminished tail current (Figure 5C) and a positive shift in channel availability (Figure 6C). However, in contrast to N588K mutation where the channel inactivates only at a more positive potential than the physiological potential range of phase II repolarization of the cardiac action potential [34,35], the H1153Y mutant induces only a modest positive shift of the half-inactivation, thus as its inactivation occurs at more negative potential than the phase II repolarization potential, indicating a little influence on this phase. Hence, the dominant changes for the H1153Y mutant is its reduced tail current corresponding to a loss of function that may induce a reliable decrease of the phase III repolarization phase of the cardiac action potential leading to a long QT phenotype. 

Whether these channel dysfunctions are enough to provide sudden death is debatable, and other factors may have influenced the severity of the disease. The role of activity as a trigger of cardiac events in LQT2 patients has already been described [36] and is very likely in our SADS case. Additionally, genetic factors such as polymorphisms [37] or non-genetic factors such as extracellular acidosis [38] or autonomic and neurohormonal factors cannot be excluded.

While our key data obtained with the combination of several technological approaches strongly support the correlation of the H1153Y variant to the SADS case investigated, the present study does not, however, pretend to solve all questions. Nevertheless, we believe that the present report may constitute a major step forward to prompt future studies and collaborative works that will certainly help to address some limitations we would like to point out. Firstly, the family history and ECG records were not obtained. As a result, a deeper analysis of the association between the clinical phenotype, genetic findings, functional data, and the family history of syncope or SCD was limited.

Secondly, these data, obtained by in vitro experiments using HEK293 cells, do not exactly duplicate the physiological in vivo environment. Furthermore, we did not examine the potential impact of accessory proteins such as the KCNHE2 channel [39] or ß-adrenergic modulation via cAMP [40]. Therefore, the characterization of H1153Y in more complex and complete physiological models such as transfected cardiomyocytes should be performed to evaluate the impact on action potential duration. Taking into account the postmortem delay of a few days between the death and the autopsy, the approach using human induced pluripotent stem cells, largely employed in LQT patient cohorts, is unfortunately very limited in the postmortem context.

To conclude, this study characterized the H1153Y variant identified in a SADS victim and showed that hERG-H1153Y is a loss of function mutation that may cause a reduction in hERG current densities in a heterologous expression system without inducing Kv11.1 channel-disrupting trafficking. These results constitute a major step forward that could be completed by further functional studies to confirm the molecular pathogenesis of the H1153Y-*KCNH2* mutation in order to discover appropriate treatment and preventive measures for LQTS patients.

## 4. Material and Methods

### 4.1. Case Description

As we have previously reported [14], a 20-year-old male who died during a bicycle exercise in a fitness room was autopsied to determine the cause of death. His medical history was unremarkable. No ante-mortem ECG was available, and the segregation family was not feasible (no answer could be obtained after contacting the family). Gross examination and histologic examination of the heart (myocardium, coronary arteries, valves, conduction system) revealed no significant abnormalities. Postmortem drug screening (ethanol, volatiles, pharmaceuticals, and illegal drugs) was negative. Molecular genetic investigations were performed from genomic DNA extracted from heart samples collected before fixing the heart in formalin.

### 4.2. Genetic Analysis

Genetic investigations were performed within the judicial mandate, which is to determine the cause of death. Targeted sequencing (Ion Torrent^TM^ PGM platform, Life technologies, Guilford, CT, USA) of 23 genes reported at the time of panel construction to be associated in the literature with inherited cardiac channelopathies was performed as reported previously [14]. Variant identification was confirmed by direct sequencing with an automated capillary sequencer. Pathogenicity of variants was determined according to the current American College of Medical Genetics (ACMG) guidelines [41].

### 4.3. Plasmid Construct, Cell Cultures, and Transient Transfection in HEK293 Cells

The chemically synthesized WT-*KCNH2* cDNA was subcloned into a pcDNA3.1-N-eGFP expression vector (Eurogentec S.A, Serain, Belgium). The missense mutation consistent with the variant detected in the proband (H1153Y-*KCNH2*) was created in the pcDNA3.1-N-eGFP-WT-Kv11.1 plasmid by a site-directed mutagenesis strategy (Eurogentec S.A, Serain, Belgium). The resultant plasmid DNA was checked by direct sequencing.

HEK293 cell line was used as a proof-of-concept unexcitable somatic cell source based on its very low levels of endogenous voltage-dependent membrane currents, and was extensively used as a heterologous expression system for studies of ion channel function [42].

Briefly, HEK293 cells were grown at 37 °C under a 5% CO_2_ atmosphere in Dulbecco’s modified Eagle’s medium (DMEM) supplemented with 10% heat-inactivated fetal calf serum. HEK293 cells with confluence ranging between 70 and 90% were transiently transfected with 5.25 μg of either construct (pcDNA3.1-N-eGFP, pcDNA3.1-N-eGFP-WT-Kv11.1, or pcDNA3.1-N-eGFP-H1153Y-Kv11.1) using Lipofectamine^®^2000 (Invitrogen, Luzern, Switzerland) according to the manufacturer’s instructions. Six hours post-transfection, the medium was replaced with DMEM supplemented with 10% FBS. The transfection efficiency was about 50–60% as assessed by manually counting the fluorescent cells expressing GFP protein after 24 h. To promote cell attachment for immunofluorescence staining and microscope imaging, cells were plated onto Labtek plastic slides 24 h post-transfection and replaced in an incubator. The different analyses were performed 48 h post-transfection.

### 4.4. Immunofluorescence Staining and Microscope Imaging

For immunostaining, the cells were fixed in 4% formaldehyde in phosphate buffered saline (PBS) 0.1 M for 10 min. After washing, permeabilization (Triton 0.1% in PBS) for 10 min and blocking buffer (5% goat serum in PBS) for 30 min were performed. Primary antibody (rabbit anti-potassium channel Kv11.1 extracellular (1:100; P0749; Sigma-Aldrich, Saint Louis, MO, USA) was added in blocking buffer and incubated for approximately 2 h at 4 °C. Cells were washed three times in PBS before incubation with goat anti-rabbit antibody conjugated to Alexa Fluor 647 (1:400; BioLegend, San Diego, CA, USA) for 1 h at room temperature (RT) and subsequently washed with PBS. Cell nuclei were stained with DAPI (4′,6-diamidino-2-phenylindole, 1/30,000 in water) for 5 min. Glass coverslips were finally mounted on microscopy glass slides using a polymerizing medium (FluorSave^TM^ reagent Calbiochem^®^-Merk, Darmstadt, Germany). For cell fluorescence acquisition, images were acquired with a Zeiss Microscope (Axio Image M2 equipped with XCite 110 LED lamp, Zeiss, Jena, Germany) and images were captured using Zen (Zeiss, Jena, Germany) software.

### 4.5. Flow Cytometry Assay of Surface Kv11.1 Channel

Briefly, 48 h post-transfection, the cells were washed with PBS and removed from the culture dish with Cell Dissociation Solution Non-enzymatic 1x (Sigma C5914; Sigma-Aldrich, Saint Louis, MO, USA). They were then incubated with rabbit anti-potassium channel Kv11.1 extracellular antibody (1:16; Sigma P0749; Sigma-Aldrich, Saint Louis, MO, USA) for 30 min at 4 °C, rinsed twice with PBS, and incubated with goat anti-rabbit antibody conjugated to Alexa Fluor 647 (1:400; Abcam 150087, Cambridge, UK) for 30 min at 4 °C. Next, cells were washed twice with PBS and resuspended in 300 µL PBS. For dead cell exclusion, cells were incubated with 0.5 µg/mL of 7-aminoactinomycin D (7AAD, Thermofischer, Waltham, MA, USA) viability staining solution for 5 min at RT. The labeled samples were analyzed using Accuri C6 Plus flow cytometry (BD Biosciences, Seattle, WA, USA).

### 4.6. Western Blot Analysis and Stain Free Quantification

Proteinase K treatment of cells was performed before protein extraction as previously described [27]. Briefly, WT and H1153Y-expressing cells plated on 6-well plates (Falcon, Corning Life Sciences, Tewksbury, MA, USA) were washed at 48 h post-transfection with PBS and incubated with 230 μL of buffer containing 10 mM HEPES, 150 mM NaCl, 2 mM CaCl_2_ (pH 7.4) with or without 200 μg/mL pK (Sigma-Aldrich, Saint Louis, MO, USA) at 37 °C for 30 min. The pK activity was stopped by adding 150 µL of ice-cold PBS containing 6 mM phenylmethylsulfonyl fluoride (PMSF) and 25 mM EDTA. The cells were then harvested and washed three times with ice-cold PBS. After incubation on ice for 1 h in a RIPA lysis buffer supplemented with complete protease cocktail inhibitor (Roche, 1X), the cell lysates were clarified by centrifugation at 4 °C for 10 min at 12,000× *g* and the supernatant was collected for protein analysis and Western blotting. Total protein concentration was determined by the BCAssay kit (Sigma-Aldrich, Saint Louis, MO, USA). Western blot experiments were conducted according to manufacturer’s instructions and as previously described [43,44]. Briefly, Western blots were carried out using 7.5% mini-PROTEAN TGX Stain-free gels (Bio-Rad, Hercules, CA, USA) loaded with 20 to 30 μg of protein lysate and subsequently transferred to polyvinylidene fluoride (PVDF) membranes (Bio-Rad, Hercules, CA, USA) using a Trans-Blot Turbo apparatus (Bio-Rad, Hercules, CA, USA). The membranes were incubated over night at 4 °C with primary rabbit anti-potassium channel Kv11.1 intracellular antibody (1:100; Sc-20130; Santa Cruz Biotechnology, Dallas, TX, USA). The blots were then washed three times for 5 min with TBS 0.1% Tween 20 (TBST) and incubated at room temperature for 1 h 30 min with horseradish peroxidase-conjugated anti-rabbit secondary antibody (1:5000, Abliance, Compiègne, FR, BI2407). The membranes were again washed with TBST three times for 5 min, incubated with Clarity chemiluminescence substrate (Bio-Rad, Hercules, CA, USA), and imaged on the ChemiDoc MP (Bio-Rad, Hercules, CA, USA). Then, the bands were detected and analyzed with ImageLab 4.1 software (Bio-Rad, Hercules, CA, USA) and normalized to the stain-free membrane.

### 4.7. Electrophysiological Recording

For electrophysiological characterization, heterologous expression of *KCNH2* was performed in HEK293. Cells were seeded at 10^5^ cells per 35 mm culture dish. Plasmid construct containing WT- or mutant H1153Y-*KCNH2* insert was transfected into HEK293 cells using lipofectamine as described above. As H1153Y mutation form in the patient is heterozygous, in some experiments, co-expression of WT- and H1153Y-*KCNH2* was performed in the same conditions using 5.26 µg of a mixture of equal amounts of each plasmid construct. Patch-clamp recording was performed 24 h after transfection.

Whole-cell currents were measured using an Axopatch-200B amplifier (Axon Instruments, San Jose, CA, USA) in its voltage-clamp mode in conditions of NaCl bath solution (in mM: NaCl 150, KCl 5, CaCl_2_ 2, MgCl_2_ 2, HEPES 10, and D-glucose 10; pH adjusted to 7.4 with NaHO) and KCl pipette medium (KCl 140, NaCl 5, CaCl_2_ 1, EGTA 5.5, MgCl_2_ 2, and HEPES 10; pH adjusted to 7.2 with KOH). Current signal was low-pass filtered at 2 kHz before a 5 kHz digitization using a Digidata 1322A card interface (Axon Instruments, CA, USA) and Pclamp software package (Axon instruments, San Jose, CA, USA).

Reversal potential determination was performed using a step potential protocol depolarizing to 40 mV for 800 ms and returning to −140 mV for 5 ms for reactivating hERG channels and then stepping to potentials from −140 to 40 mV; the instantaneous current was plotted against these potentials. The I–V distribution was almost linear and reversal potential was estimated by linear regression.

Data were analyzed offline using the Clampfit routine of the Pclamp 10.7 software package. Conductance (G)–voltage (V) relationships were constructed after leak subtraction using passive current at membrane potentials more negative than −50 mV. Classical G–V relationships were analyzed using the Boltzmann model of the following form:

$$\left(V\right)=\frac{Gm}{1+e\left(\frac{V_{1/2}+V}{k}\right)}+c$$

where *Gm* is the maximal activated conductance, *V*_1/2_ is the half maximal activating potential, and k is the slope factor.

Activation time (t) course was analyzed using an exponential power model using the following equation:

$$f\left(t\right)=\overset{n}{\underset{i=1}{\sum }}A_{i}{\left(1-e^{-t/\tau_{i}}\right)}^{a}+c$$

where *A_i_, τ_i_*, and *c* have the classical meanings. Deactivation current was analyzed using a standard exponential function of the following form:

$$f\left(t\right)=\overset{n}{\underset{i=1}{\sum }}A_{i}e^{-t/\tau_{i}}+c$$

Nonlinear mathematical model fitting to data was performed using Clampfit, and estimated parameters are given as mean ± SEM.

### 4.8. Statistical Analysis

The data were analyzed using Microsoft Excel and Prism6 (Graph Pad software, San Diego, CA, USA). Continuous variables are presented as mean ± standard error of the mean (SEM). Analysis using D’Agostino–Pearson normality test indicated that the data obtained were not distributed normally. Consequently, the data were analyzed using the nonparametric Mann–Whitney U test. A *p*-value less than 0.05 was considered statistically significant. For electrophysiological data, statistical significance of the difference between means was determined using either unpaired *t* test statistics or one-way ANOVA followed by Newman–Keuls multiple comparison tests using Graph-Pad Prism5.

## Figures and Tables

**Table 1:** Molecular genetic characteristics of causal variant H1153Y.

DNA Change (cDNA)	Protein	Frequency of Variant (GnomAD)	Mutation Tester	SIFT Prediction	Polyphen-2	Previously Reported in Patients (HGMD) and Phenotype	ACMG
c.3457C > T	P.H1153Y	0.0048%	Disease causing	Deleterious	Probably damaging (0.981)	Yes/LQTs [22]	3 [24]

**Table 2:** Electrophysiological properties on current density of C-term *KCNH2* variants with no trafficking deficiency.

Study	Phenotype	Variant	Current Density
			pA/pF
This study	SADS	WT	58.3 ± 14.6 (19)
		H1153Y	18.7 ± 5.1 * (23)
		WT + H153Y	55.7 ± 20.4 (8)
Anderson et al., 2014 [18]		WT	97.7 ± 6.2 (9)
	LQT2 patient (0–90 y.o)	R1005Q	97.7 ± 6.2 (7)
	LQT2 patient (0–90 y.o)	L1049P	34.6 ± 4.5 (7)
	LQT2 patient (0–90 y.o)	L1066V	74.3 ± 8.7 (6)
Biliczki et al., 2008 [20]		WT	8.0 ± 1.0(13)
	Macrolide-induced QT prolongation, asymptomatic	R954C	4.2 ± 1.2 * (5)
	Survived SCD, no syncope	G1036D	6.3 ± 1.4 * (7)
Anson et al., 2004 [33]		WT	94.4 ± 18.3 (19)
	SNP in SIDS cohort	K897T	57.3 ± 7.2 (22)
	SNP in SIDS cohort	P967L	116.9 ± 18.3 (20)
	SNP in SIDS cohort	R1047L	129.5 ± 14.5 (20)
	SNP in SIDS cohort	Q1068R	126.1 ± 13.9 (20)

Values with an asterisk indicate statistical significance (*p* < 0.05) compared with WT; SADS, sudden arrhythmic death syndrome; SCD, sudden cardiac death; SIDS, sudden infant death syndrome; SNP, single nucleotide polymorphism.

## Data Availability

Additional data are available from the corresponding author on request. No data are deposited in databases.
